# Compassionate Love as a Predictor of Reduced HIV Disease Progression and Transmission Risk

**DOI:** 10.1155/2013/819021

**Published:** 2013-11-18

**Authors:** Heidemarie Kremer, Gail Ironson, Lauren Kaplan, Rick Stuetzle, Mary A. Fletcher

**Affiliations:** ^1^Department of Psychology, University of Miami, FL, USA; ^2^Department of Medicine, University of Miami, FL, USA

## Abstract

*Objectives*. This study examined if compassionate love (CL) predicts HIV disease progression and transmission risk. Scientific study of CL emerged with Underwood's working model of other-centered CL, defining five criteria: free choice, cognitive understanding, valuing/empowering, openness/receptivity for spirituality, and *response of the heart. Method*. This 10-year cohort study collected 6-monthly interviews/essays on coping with HIV and trauma of 177 people with HIV in South Florida. Secondary qualitative content analysis on other-centered CL inductively added the component of CL towards self. Deductively, we coded the presence of the five criteria of CL and rated the benefit of CL for the recipient on a 6-point Likert scale. Growth-curve modeling (reduced to 4 years due to cohort effects) investigated if CL predicts CD4 slope (HIV disease progression) and cumulative viral load detection (transmission risk). *Results*. Valuing/empowering and cognitive understanding were the essential criteria for CL to confer long-term benefits. CL had a higher benefit for recipients if given out of free choice. High scores of CL towards self were reciprocal with receiving (93%) and giving (77%) other-centered CL. Conversely, those rated low on CL towards self were least likely to score high on receiving (38%) and giving (49%) other-centered CL. Growth-curve modeling showed that CL towards self predicted 4-year cumulative undetectable viral load (independent from sociocultural differences, substance use disorder, baseline CD4 and viral load). Those high versus low on CL self were 2.25 times more likely to have undetectable viral load at baseline and 1.49 times more likely to maintain undetectable viral load over time. CL towards self predicted CD4 preservation after controlling for differences in CL giving. *Conclusions*. CL towards self is potentially the seed of being expressive and receptive of CL. Health care professionals prepared to walk the extra mile for those who neglect and isolate themselves may break a vicious circle since those lacking CL self were least likely to receive CL from others. Future studies should examine whether any enhancement of CL towards self may translate into slower disease progression and reduction of transmission risk.

## 1. Introduction

A couple of decades ago, research on compassionate love (CL) seemed “conspicuous by its absence” (p. 2) [1]. Medical research in this area is scarce. Entering the keywords compassionate love in PUBMED yields only 24 publications, four of which address HIV care, since the topic emerged with the AIDS epidemic. The first PUBMED listed publication is from Uganda in 1989, in which Nelson Sewankambo opined that CL may be the essential clinical skill to be trained to improve the quality of life of people living with HIV (PLWH) [2]. Ten years later, the term surfaced as a topic of scientific interest in the US after a 1999 conference at the Massachusetts Institute of Technology brought together several key theorists and researchers to edit a compendium on altruistic love [3]. CL is closely related to but not synonymous with concepts such as altruistic love, unlimited love, romantic love, unconditional love, and agape (p. 72) [3, 4]. In the World Health Organization (WHO), the term first emerged on World AIDS Day 1994, when the Director General of the WHO called upon families of human kind (including health and spiritual caregivers, people living with HIV and their advocates and networks) together to give compassionate care and love to PLWH [5]. The WHO summoned health professionals with both religious and nonreligious backgrounds in a working group, on the role of spirituality in quality of life, to develop a cross-cultural quality of life assessment tool for PLWH [6]. Cultural discordances emerged as the Buddhists preferred the term “compassion” instead of “love” and the Muslims argued that compassion was too “cold” and that “love” was the necessary term, “compassionate love” was the compromise term to portray the spiritual aspect of quality of life (pp. 8-9) [4].

Since 2001, the scientific study of CL has received support from the Fetzer Institute, and the study presented here is one of the selected projects made possible through this funding [7]. Based on interviews with Trappist monks [8], Underwood defined the term CL as “love that centers on the good of the other.” CL can be expressed through in attitudes, actions, words, and body language, including altruism, helpfulness, genuine concern, empathy, and other elements that are commonly shared by the diverse concepts of love [8]. Furthermore, Underwood defined five basic criteria of CL: *free choice* for the other; some degree of accurate *cognitive understanding* of the situation—the other and oneself; *valuing/empowering* the other at a fundamental level; *openness and receptivity for spirituality *and *response of the heart* [8]. Detailed definitions and examples of those five criteria of CL are depicted in Table 1. It is unclear if those criteria are all essential for CL to be expressed, and it is unknown how much each criterion contributes to the benefit of CL for the recipient [3, 4, 8]. 

**Figure 1:**
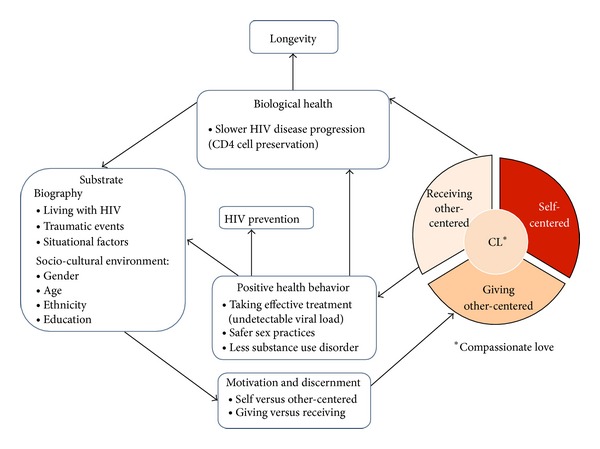
Compassionate love (CL) and health in people living with HIV (PLWH). The framework of our study is based on Underwood's working model of CL (italic font) (p. 76) (p. 76) [3] and our analysis of the individual biographies of 177 PLWH. Using hierarchical linear modeling [33], we test if CL (giving, receiving, and self) predicts positive health behaviors preventing HIV disease progression and transmission, contributing to better biological health, independent of sociocultural factors. A survival analysis examining the link between CL and longevity will follow [17].

Our structural framework of “Compassionate Love and Health in People with HIV” as illustrated in Figure 1 is based on the “Working Model of Compassionate Love” of Underwood (p. 76, italic fonts in Figure 1) [3], supplemented by our own HIV research and findings of others. According to Underwood (p. 76) [3], the motivation and discernment to express CL are based on the individual variations in the person's personality, biology, development, and situational factors, nested within and shaped by the sociocultural environment. CL increases the possibility of positive behavior, resulting in a positive feedback loop changing the substrate of CL, which is constituted by the individual biography and the sociocultural environment. Conversely (not shown in Figure 1), inappropriate or no actions of CL result in negative behavior limiting the person's ability of expressing CL. Underwood also emphasized that CL can be expressed by making space for others or the “divine” to give (p.76) [3], which can in turn foster a person's capacity to express love to others (p. 75) [3]. According to her, CL is a dynamic process that involves feedback loops in which the expression of CL can expand the capacity to love of both the person giving and the person receiving. Hence, the dynamic process of CL can best be examined in a longitudinal design. 

For example, our longitudinal research in PLWH showed that caring behavior for others (rated from essays about stressful or traumatic situations) predicted slower HIV disease progression as indicated by biological markers, such as CD4-cells and viral load (VL), and giving to charities predicted better VL control over two years, even after controlling for baseline differences on CD4 and VL counts [9]. In addition, a study of Ramirez-Valles and Brown [10] suggests that volunteering in AIDS organizations results in an increase in self-esteem, sense of empowerment, and safer sex behaviors in people with HIV.

CL can also be received from a personal relationship with a Higher Power/God. For example, our own research showed that individuals that felt “chosen by a Higher Power” to have HIV were more likely to view their HIV diagnosis as a spiritual transformation that changed their attitudes, beliefs, behaviors, and their spirituality towards a life fulfilled with CL [11]. In addition, people who viewed God as loving and felt that God loved them had slower disease progression [12]. Conversely, people who viewed God as harsh punishing, and unforgiving had faster disease progression, even after control for baseline CD4 and VL counts, health behaviors, mood, coping, and social support [12]. Additionally, individuals viewing HIV as a punishment of God may perceive themselves as unworthy, needless, unloved, and rejected [11, 13, 14], which in turn may foster isolation, leading to a vicious circle of limited opportunity to express and feel CL.

At the center of the model are motivation and discernment (see Figure 1). For example, if someone who is HIV-positive joins a volunteer organization with the ultimate aim to get back to work despite HIV, it would not count as an act of giving CL to others, since the motive is not centered on the good of the other. Joining a volunteer organization may result in positive health behavior, such as giving up drugs/alcohol or better medication adherence and improved health, which is in turn a substrate of CL. CL involves discerning and balancing competing factors to prevent well-intentioned actions that would harm the other or oneself. For example, someone in an HIV-discordant partnership has to weigh many competing factors in family planning related to one's own health and the health of the partner and child. According to Seelig and Rosof [15], CL towards others can be completely altruistic and an act of selflessness, self-sacrifice (and thus self-injury), and it can be selfish because one may expect something, yet CL also may mean also to respect the own capacities and to “see” the others as they are.

Underwood's definition of CL is confined to giving and receiving other-centered CL (p. 75-76) [3]. However, the inductive data analysis of the first twenty interviews of PLWH discovered that compassionate self-love is a core component of the full expression of CL. As Underwood suggested, discerning the appropriate expression of CL requires us to balance our own needs with those of the other (e.g., putting the oxygen mask on oneself in an airplane emergency maximizes the benefit to self and others) p. 15 [4]. This balance dovetails with the biblical principle of “loving one's neighbor as oneself.” In other-centered selfless CL, a power balance between giving and receiving is essential to prevent giving from becoming a “one-way-street,” whereas in self-centered CL giving and receiving is always balanced.

Based on this structural framework (Figure 1), the purpose of the present study is to examine the five criteria of CL in all the components (giving, receiving, and self) to determine whether CL translates into positive health behavior to prevent HIV disease progression and transmission risk. Analyzing our ten-year collection of interviews and essays [9, 12, 16, 17], we provide refined definitions in an inductive and deductive approach using qualitative content analysis. We contribute anchor examples for each of the five criteria of CL and establish their frequency and their contribution of the benefit to the self or others. Using exploratory analysis, we calculate the extent to which each component of CL contributes to the full expression of CL. Positive health behavior is indicated by safer sex, absence of substance use disorder, and achieving an undetectable VL. Since achieving an undetectable VL requires >95% adherence and reduces HIV transmission by 96%, we use this parameter as a biological indicator of adherence as well as reduced transmission risk [18]. Slower HIV disease progression is measured by immune preservation as indicated by the CD4 cells changes over time. We test our predefined hypothesis, as postulated in Figure 1, that each component of CL is related to long-term health-preventive behavior (taking effective treatment to achieve undetectable VL, lower risk of substance use disorder, and safer sex behavior) resulting directly and indirectly into slower HIV-disease progression (CD4 cell preservation) and lower HIV transmission risk (cumulative undetectable VL). Finally, we discuss the clinical implications of our results that may be applicable to other diseases as well. Another future analysis will examine the association between CL and longevity (in preparation).

## 2. Method

 In our longitudinal study on the psychosocial and spiritual factors related to health and longevity with HIV, we followed 177 people with HIV intensively for up to ten years [12, 15, 16]. In this secondary data-analysis, we transcribed one interview of the most traumatic time point within the first three years of study entry and summarized all other available interviews and essays of the 177 participants. We used a mixed method approach, applying qualitative content analysis (inductive and deductive) to code the presence of the five criteria of CL within all three components (giving, receiving, and self) and to rate the benefit of CL for the recipient on a 6-point Likert scale. Quantitative variables were then derived from frequencies of these codes and ratings. Hierarchical regression analysis determined the explanation of variance of the full expression of CL for CL giving, receiving, and self. Growth-curve modeling, a type of hierarchical linear modeling, tested our working model (see Figure 1) to examine if CL (giving, receiving, and self) predicts CD4 cell preservation and cumulative undetectable VL. Although we have up to ten years qualitative data, we were only able to predict the biological outcomes over four years due to the cohort effect.

### 2.1. Study Population and Sampling

From 1997 to 2000, our study recruited 177 people with HIV via flyers (distributed through doctors' offices, community events, support groups, HIV organizations), newspaper ads, and word of mouth. Participants were diverse with respect to gender (30% female), age (mean age 37.49 ± 8.88 years), ethnicity (36% African American, 31% White (non-Latino), 28% Latino), and sexual orientation (45% heterosexual). Despite 68% attending further education beyond high school, most participants were relatively poor with 60% living on less than $10k annual income, which is consistent with their high unemployment (15%) and disability (42%). The entry criteria selected those in the midrange of HIV disease (150–500 CD4-cells/mm^3^, CD4-nadir >75 cells/mm, no prior AIDS-defining symptoms or HIV associated dementia), because we hypothesized that psychological factors would have maximum impact at this disease stage. To reduce unreliability or confounders for immune response, we excluded people with active substance use disorder and/or psychosis (based on the Structured Clinical Interview, DSM-III-R [19]).

### 2.2. Procedures

The study was IRB approved; all participants gave informed consent and received $50 compensation per visit. From 1997 to 2007, we collected in-depth data interviews and essays in six-month intervals, asking how they cope with HIV and other life traumas as well as their safer sex practices. At every time-point, participants completed a comprehensive set of questionnaires including sociodemographics and health behaviors and gave blood and urine samples for biological measurements [12, 15, 16].

Transcriptions and in-depth qualitative data analysis took place from 2009 to 2012. We fully transcribed the interview of the time-point with the highest negative rating (closest to −3, very stressful) on the life-event scale [20, 21] during the first three years of study which also served as the basis of our analysis of spiritual coping with trauma [18, 22]. Beside this interview/essay, a team of ten trained transcribers summarized the content of all other interviews and essays for up to ten years. For some participants, more than one interview was fully transcribed if it contained relevant information for inductive coding. All transcripts were quality controlled, entered in the qualitative software atlas.ti, and rated using directed qualitative content analysis [23–25]. For further statistical analysis, the 15 codings and three ratings were aggregated and transferred into SPSS version 19. HLM software version 6.03 was used for the hierarchical linear modeling. 

#### 2.2.1. Interviews and Essays

In the initial interview, we explored how participants believed they got HIV and how they reacted to their diagnosis. At the initial and every follow-up interview, we asked to whom they disclosed their HIV status (and if gay, their sexual orientation) and how the reaction was; how their life changed since the diagnosis; how they spent their daily life; which activities they looked forward to; if they had a partner, were sexually active, practiced safer sex, and disclosed their HIV status to their partners; if and how their partners were helpful to them and if they had someone to take care of them if needed and to share their deepest feelings with; and if they had partners or friends who died from AIDS and how they reacted. Other questions tapped into their spiritual beliefs, beliefs about death and dying, afterlife, and life expectancy. We asked how they found their physician and if they were satisfied with their medical care; what they were doing to keep themselves healthy; what percentage of their well-being was due to their own attitudes and behaviors versus medical care; if they were getting complementary or alternative treatment; and if they were taking prescribed medication and the reason behind it. Finally, we asked about what enabled them to keep going in the face of HIV and if anything positive had resulted from being HIV-positive or anything else was relevant to maintaining their health in the face of HIV. In addition, interviews and essays captured how they coped with the most difficult life event over the past six months.

### 2.3. Qualitative Content Analysis of Compassionate Love

Figure 1 describes theoretical framework of our content analytic approach. Each time a full transcript of one or more interviews and a detailed summary of all other essays/interviews was completed, the transcriber worked with the research team inductively line by line through the entire transcript to highlight quotes and anchor examples. The text passage quote was the smallest unit of our analysis to develop a categorization system. Deductively, we coded the five criteria of CL derived from Underwood's basic theoretical mode of CL [3, 4, 8]. Inductively, we searched for additional components of CL and a method to rate the benefit of CL for the recipient.

After analyzing 20 transcripts, inductive coding revealed that CL towards self was an additional component of CL that should be included in the ratings (see Section 1). After more than 50 interviews, we reached a point of saturation and compiled tentative definitions for the coding of the presence of the five criteria for each component of CL, giving, receiving and self. Each interview underwent 15 codings, grounded in at least one characteristic quotation, which are explained in Table 1. Further, we developed a 6-point Likert scale rating for the benefit of CL for the recipient (ranging from 1 = CL *negative or harmful* to 6 = CL is* a central element in the person's life*), which was based on the overall coding of the transcript.  Table 2 explains the rating of the benefit of CL for the recipient with characteristic anchor examples for CL (giving, receiving, and self). The main purpose of Tables 1 and 2 was to provide an explicit coding agenda with definitions and examples to illustrate under what circumstances a quoted text passage can be coded/rated [24, 25].

Next, ten raters established independent interrater reliability by coding and rating the first twenty interviews. For each Cronbach's alpha < .80, definitions were revised. Using the revised versions, two raters established chance-corrected interrater reliability for additional 20 interviews. The fine-tuned definitions of the codes of the five criteria had substantial reliability (Cohen's Kappa above .60), and reliability of the rating of the benefit of CL was excellent (Kendall's tau *B* = .81, *P* < .001) [26]. Finally, all 177 transcripts were fully analyzed independently by two raters and any discrepancies were either consensually agreed or discussed in the team. For any rating with discrepancies between two independent raters that was greater than one point on the 6-point Likert scale, another pair of raters performed again an independent coding and reviewed discrepancies consensually, if they occurred. In case there was not enough information for a reliable coding/rating, we excluded the case from further analysis. Not enough information applied to four codings of the criteria of CL (involving 2 *response of the heart* and 1 *openness/receptivity for spirituality *in CL self, and 1 *cognitive understanding* in CL giving) and to 20 ratings of the benefit of CL (involving 14 CL giving, 2 CL receiving, and 4 CL self). In total, we categorized 177 transcripts into 15 codes (presence of 5 criteria of CL for each of the 3 components) and three Likert scale ratings (benefit of each of the 3 components of CL for the recipient) providing one or more text passage quotes for each coding/rating.

#### 2.3.1. Biological Measurements

As an immunological indicator of HIV disease progression, we measured CD4-cells/mm^3^ with whole blood four color direct immunofluorescence, utilizing a coulter XL-MCL flow cytometer. For VL testing, we used the Roche Amplicor RT/PCR assay, sensitive to 400–750,000 copies/mL. Since an undetectable VL reduces HIV transmission risk by 96% [27], we used this parameter also as a dichotomous biological indicator of HIV transmission risk. Seven of 177 people were excluded from the growth curve modeling, since they did not provide a second blood sample at their follow-up time-point. Due to the cohort effect (loss of followup due to death, health deterioration, relocation of participants), 66/177 (37%) remained in the study after four years. This only allowed us to predict biological and clinical measures of health and health behavior over four years. 

#### 2.3.2. Health Behavior Measurements

In order to achieve and maintain an undetectable VL, greater than 95% adherence is required [28–31]. Originally, we measured self-reported treatment adherence of less than 95% over the past three days using the interviewer-administered adherence questionnaire of the AIDS Clinical Trials Group [32]. Data quality control revealed that the responses were subject to self-report bias, overestimating adherence. Of the 134 participants taking antiretroviral therapy at baseline, 63% had a detectable viral load but only 26% reported less 95% adherence over the past three days. Therefore, cumulative undetectable VL over four years was also used as a dichotomous biological indicator of taking and adhering to effective HIV treatment [18]. Safer sex behavior averaged over four years was based on the interview responses on the frequency of sexual activities, use of protection, number and HIV status of the partners. Based on this data, we calculated the percentage of those reporting having unprotected sex, consistent safer sex practices, and sexual abstinence over four years. Applying the Structured Clinical Interview [19], we controlled for substance use disorder onset/relapse over four years for all but six participants.

### 2.4. Statistical Analysis

Descriptive statistics were calculated for the coding of the five criteria, the ratings of CL (giving, receiving, and self), health behaviors, and health parameters. Likelihood ratio (LR) tests examined the association between each component of CL. Hierarchical regression analysis with stepwise deletion determined the importance of the five criteria in explaining the variance of each CL component and of the three components in explaining the variance of the sum of the CL components. Independent Student's *t*-test compared mean values of CL among those with sexual risk behavior and substance use disorder over four years. Hierarchical linear modeling [33], specifically growth curve analysis, tested our hypothesis that CL translates into CD4 cell preservation and cumulative VL over four years. The base model was composed of two levels: at level 1, equations were used to model the intraindividual changes in CD4 cells and VL over time, while at level 2, equations modeled inter-individual differences in CL, age, gender, ethnicity, education, and baseline CD4 and VL status. CL was added last so that the significance of CL as a predictor was estimated after controlling for differences in sociocultural and baseline biological parameters. Bernoulli estimation models were used for the prediction of achieving/sustaining an undetectable VL. We examined post hoc if associations/trends between CL towards self and viroimmunological outcome remained/became significant after controlling for substance use disorder, VL slope, CL giving, and CL receiving.

## 3. Results

After an overview of the coding of the five criteria of CL and the rating of the effect of each component of CL, we present the results of our regression analysis and hierarchical linear modeling, testing our hypothesis for the direct and indirect links between CL and biological health outcomes. 

### 3.1. Five Criteria of CL

**Figure 2:**
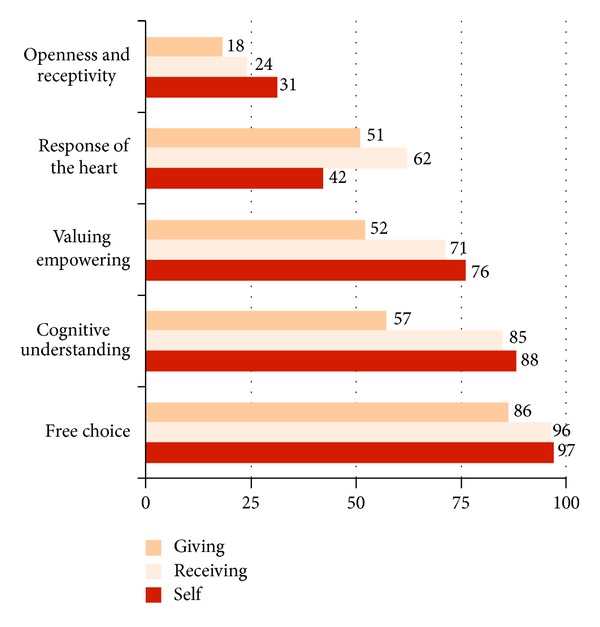
Presence of the five criteria (free choice, cognitive understanding, valuing/empowering, response of the heart, and openness and receptivity for spirituality) for all three components of compassionate love: giving, receiving, and self (% frequencies of the 15 codings detailed in Table 1).

Table 1 defines the five criteria of CL with illustrating examples for each component. As depicted in Figure 2, free choice is the most frequent criterion coded, followed by cognitive understanding, valuing/empowering, and *response of the heart*. Openness/receptivity *for spirituality* is the least present criterion. Over all components, the frequency order follows a pattern, with every criterion being most present in CL self, followed by CL receiving and CL giving, except for *response of the heart*, which is most common in CL receiving and least in CL self.

### 3.2. Rating of the Components of CL

**Figure 3:**
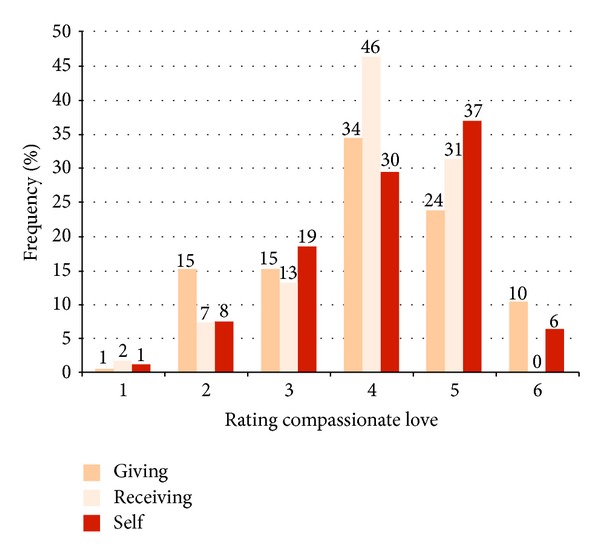
Compassionate love giving, receiving, and self (% frequencies of the 3 ratings detailed in Table 2). The rating is on a 6-point Likert scale with 1 = CL negative or harmful, 2 = no expression of CL, 3 = CL attempted but not beneficial, 4 = CL beneficial but not empowering, 5 = CL beneficial and empowering, and 6 = CL is a central element in the person's life.

Table 2 explains the rating of the benefit of CL for the recipient with characteristic anchor examples for CL (giving, receiving, and self), and Figure 3 shows the percent frequencies of the rating on a 6-point Likert scale. On average, CL was rated high (*M* = 4.13 ± 1.10 for CL towards self, *M* = 3.98 ± 0.95 for CL receiving, and *M* = 3.97 ± 1.22 for CL giving). Overall, beneficial CL (range 4–6) was rated by 73% for CL self, 69% for CL giving, and 78% for CL receiving.

Grouping CL at the 25th versus 75th percentile compares participants with low scores (nonbeneficial, no or even self-damaging CL, score = 1–3) versus those with high scores (self-empowering CL or even CL as a core element in the person's life, score = 5-6). High CL towards self had the strongest association with high CL receiving (LR 54.06, *P* < .001). Almost all participants with high CL towards self (93%) also received high CL from others, compared to only 38% of those with low CL towards self. Moreover, high CL towards self was also related to giving high CL towards others (LR 11.02, *P* = .001). Conversely, most participants with low CL for themselves (62%) received low CL from others. Most (77%) participants with high CL self also gave high CL to others, compared to 49% of those scoring low on CL towards self. Similarly, in most (88%) participants, giving high CL to others was reciprocated by receiving high CL from others, compared to 57% receiving beneficial CL from others among those low on CL giving (LR 18.84, *P* < .001). In summary, CL towards self and others was reciprocal, with those low on CL towards self being least likely to receive CL from others.

### 3.3. Hierarchical Linear Regression Analysis

To determine how much variance in CL (giving, receiving, and self) was explained by the five criteria, we employed a hierarchical linear regression analysis with stepwise exclusion. According to the adjusted *R* ^*2*^, the five criteria explained 53% of variance in CL giving (33% valuing/empowering, 10% cognitive understanding, and 10% other three criteria), 40% of variance in CL receiving (19% valuing/empowering, 11% free choice, and 10% other three criteria), and 48% of variance in CL self (29% valuing/empowering, 10% cognitive understanding, and 9% other three criteria).

Intercorrelations between the components of CL were significant (self/giving *r* = .60, self/receiving *r* = .45, giving/receiving *r* = .40; *P*'s <.001). Hierarchical linear regression calculated how much variance of the full expression of CL (indicated by the sum of CL giving, receiving, and self) was explained by each component. According to the adjusted *R* ^*2*^, CL self contributed 71%, CL giving 21%, and CL receiving 8% to the explanation of variance in full expression of CL. The correlation between CL self and the sum of CL (CL self, CL giving, and CL receiving) was *r* = .84, *P* < .001, which demonstrates that CL self is almost congruent with the full expression of CL. 

### 3.4. Longitudinal Relation between CL and Safer Sex and Less Substance Use Disorder

Over four years, 19% of the participants reported having unprotected sex, 62% stated consistent safer sex practices, and 19% reported sexual abstinence. Substance use disorder onset/relapse occurred in 29% of participants. In line with our directed hypothesis (one-tailed significance testing), independent *t*-tests showed lower mean scores on CL for those with substance use disorder (CL giving *M* = 3.65 ± 1.11 versus 4.01 ± 1.23, *P* = .017; CL receiving *M* = 3.76 ± 0.92 versus 4.05 ± 0.94, *P* = .029; CL self *M* = 3.79 ± 1.07 versus 4.25 ± 1.10, *P* = .008) but not among those with unsafe sex practices. Ratings of CL and frequency of sexual interactions, use of protection, number and HIV status of the partners were not significantly correlated.

### 3.5. Hierarchical Linear Modeling of the Link between CL and Biological Health Outcome

At baseline, only 29% of the participants had an undetectable VL indicating a poor uptake and adherence to HIV treatment at study entry. CL towards self was significantly higher among those who entered the study with an undetectable VL (CL self *M* = 4.35 ± 0.93 versus 4.03 ± 1.15, *P* (one-tailed)  = .040), whereas baseline VL was not associated with other-centered CL. Baseline CD4 cells (*M* = 296.71 ± 102.45) were not significantly correlated with self- and other-centered CL.

Hierarchical linear modeling allowed us reliable prediction of CD4 and VL slopes over four years, although the study measured CD4 and VL over ten years.  Table 3 provides an overview of CL as a predictor of CD4 cell preservation and cumulative VL over four years. Only CL towards self predicted cumulative undetectable VL, irrespective of sociocultural factors and baseline biological parameters (1-tailed *P* = .003). Since CL towards self was significantly associated with less substance use disorder (see above) we also confirmed that the association between CL towards self and cumulative undetectable VL was not mediated by substance use disorder.

Since all three components of CL were highly intercorrelated, we tested if the association between CL towards self and cumulative undetectable VL was independent of differences in CL giving and receiving. As postulated in our structural framework, controlling for CL giving, which in itself showed a tendency towards achieving cumulative undetectable VL (1-tailed *P* = .147), increased the strength of the link between CL self and cumulative VL (1-tailed *P* = .002). Hence, if all participants were equally engaged in giving CL to others, CL self would be a stronger predictor of treatment success and HIV transmission risk. In line with our hypothesis, after controlling for CL receiving, CL self was no longer significantly associated with cumulative undetectable VL (1-tailed *P* = .468). In other terms, if those low on CL self would not be less likely to receive CL from others, lower CL self would no longer predict HIV risk behavior (decrease in undetectable VL over time).

There was a trend between CL towards self and CD4 cell preservation (1-tailed *P* = .112), which was not altered after control for substance use disorder but disappeared after control for cumulative undetectable VL (1-tailed *P* = .468). Since cumulative VL predicted CD4 slope over 4 years (coefficient 1.767, *t*-ratio 2.557, df 959, 1-tailed *P* = .006), the trend between CL towards self and CD4 preservation was explained by differences in treatment success. Moreover, the trend between CL towards self and CD4 cell preservation became significant only after controlling for CL giving (1-tailed *P* = .048) and stronger after controlling for CL receiving (1-tailed *P* = .057). In other terms, CL towards self predicts CD4 cell preservation after controlling for CL giving. As noted above, PLWH giving high CL towards self were more likely to give high CL towards others. If participants would be equal in giving CL to others, CL towards self would predict a better immunological outcome.

Overall, achieving undetectable VL tended to decline over time (undetectable VL slope coefficient = −0.048, *t*-ratio = −1.780, df 161, 2-tailed *P* = .077), irrespective of sociocultural background and initial CD4 and VL status. To examine the protective effect of high versus low CL self, we calculated the ratio of the slope of change in undetectable VL for those scoring low on CL towards self (at the 25th percentile, score = 3, *n* = 45) compared to those scoring high (at the 75th percentile, score = 5, *n* = 73). Over time, undetectable VL was 1.49 times more likely among those high versus low on CL towards self (even after controlling for baseline differences in undetectable VL); in addition, they were already at baseline 2.25 times more likely to achieve undetectable VL (36% versus 16%, 2-tailed *P* = .002). The protective effect of high CL self was potentially underestimated, since we had a significant time effect (e.g., 15% had died as of 4/30/2004 and 32% as of 4/30/2010). A Chi-square test showed that more participants dropped out of the study among those low on CL self (32% versus 38%, *P* = .05). 

Thus, our hypothesis of a positive link between CL and biological outcomes of health behaviors and health was supported for CL towards self. Nevertheless, differences in giving and receiving CL alter the strength of the association between CL towards self and positive viroimmunological outcome. Notably, only 38% of those low on CL self received high CL from others, compared to 93% of those with high CL self (*P* < .001). CL towards self no longer predicted undetectable VL after controlling for differences in CL receiving. In other terms, if those low on CL self would have equally received CL from others, a higher risk of treatment failure and HIV transmission could have been prevented. In addition, giving high CL to others was more prevalent among those high versus low on CL self (77% versus 49%, *P* = .001). After control for CL giving, CL towards self predicted CD4 cell preservation. Thus, provided if participants would be equal in giving CL to others, CL towards self would predict a better immunological outcome as well.

## 4. Discussion

In this study in PLWH, we added the third component of CL towards self to Underwood's working definition of giving and receiving other-centered CL (p. 75-76) [3]. Based on a report of Doug Oman to the Fetzer Institute [1], this is the first study that is fully operationalizing all five criteria defined by Underwood (*free choice*, *cognitive understanding*, *valuing/empowering*,* openness/receptivity for spirituality*, and *response of the heart*). We analyzed how essential each criterion is for CL to be beneficial for its recipients. In addition, we examined how the benefit of self- and other-centered CL are intertwined and which component of CL predicts longitudinally into biological outcomes (CD4 cells, VL), indicating slower HIV disease progression, taking and adhering to antiretroviral therapy, and lower transmission risk. Finally, we will discuss the potential role of health care professionals in fostering CL based on these findings.

### 4.1. Essential Criteria for the Benefit of CL

According to our coding, almost all participants expressed and received CL out of *free choice*, mostly with *cognitive understanding* and *valuing/empowering*, which was beneficial in about two-thirds of the cases. The essential criterion for the perceived benefit of CL is valuing and empowering the other and oneself at a fundamental level. Additionally, *cognitive understanding* of the needs and feelings of the other and oneself is related to the perceived benefit of CL. Both criteria may be encouraged by health care professionals. When PLWH receive CL, it is more likely to be rated as effective if it is given out of *free choice*. Ironically, in the health care setting the professional duty may be a barrier to the effectiveness of CL since *free choice* was the second most important criterion for the rating of a positive effect of CL. Based on the interviews/essays, the *response of the heart* and the *openness and receptivity for spirituality* are less frequent and less essential criteria for a positive impact of CL. For clinical practice, this means that valuing and empowering PLWH is perceived as an important component of care in the health-care setting.

### 4.2. The Relation between Self- and Other-Centered CL—Clinical Implications and Applications

Interestingly, the ratings of CL towards self are highly intercorrelated with those of receiving CL from others and giving CL to others. Based on the ratings, almost all participants who took care of themselves benefited from CL from others as well. Although the direction of this association remains to be established, receiving CL from others potentially promotes better self-care. Unfortunately, those who rated low on CL towards self—and thus more in need of CL from others—were those who mostly lacked supportive CL from others. This dovetails with our prior research on spiritual transformation [34]. Potentially, people who neglect and isolate themselves are the ones who are mostly in need of receiving intrinsically motivated CL from health care professionals and are at the same time least likely to receive attention. Furthermore, those who are not receptive for CL towards self may be also less receptive for CL from others, even if provided. A study of Oman et al. [35] showed that health care professionals may enhance their self-efficacy to provide CL to patients by practicing passage meditation. Perhaps such spiritual interventions are useful to train health care professionals to meet their patients' need for CL. 

According to our rating, putting the well-being of the other in the center was reciprocated by others and coincided with better self-care. Based on those ratings, health care professionals may promote volunteering or care taking of others in PLWH to enhance both self-care and social support. Furthermore, Carson et al. [36] found that an intervention based on loving-kindness meditation focusing on both feelings of self- and other-centered love related to reduced pain, anger, and psychological distress in people with back pain. This supports the notion that health care professionals may enhance CL using spiritual interventions. 

### 4.3. CL towards Self as the Basis of Other-Centered CL

The most relevant finding of our study is that ratings of self-centered CL were almost congruent with the total amount of CL expressed (giving, receiving, and self). Based on our ratings, promoting CL towards self, experience of self-value and self-empowerment, and the cognitive understanding of one's needs are important steps to enhance the reciprocal exchange and growth of CL. Our findings support the theories of Underwood that receiving and giving other-centered CL fuels self-centered CL and vice versa (p. 75-76) [3]. The central role of CL towards self, which was identified clearly in our inductive qualitative analysis, should be added to the Underwood's working model of CL. In summary, our results suggest that CL for oneself is potentially the seed of expressing CL towards others and being receptive for CL from others. 

### 4.4. CL towards Self Translates into Healthier Behavior and Health

In line with our above results, CL towards self was the only component of CL ratings that ultimately predicted healthier behavior, lower HIV transmission risk, and a trend towards better biological health. Prior studies indicate that achieving an undetectable VL requires both receiving excellent health care (including a good patient-physician relationship), taking, and adhering HIV treatment [37]. Maintaining an undetectable VL could limit sexual transmission of HIV [27] as well as mother to child transmission [28]. Although CL was not associated with sexual risk behavior, people rated as expressing more compassionate love towards self have a lower risk of transmitting HIV to others. This is because they are more likely to achieve and sustain an undetectable VL over time, which reduces HIV transmission risk by 96% [27]. In addition, self- and other-centered CL ratings were associated with a lower risk of substance use disorder onset/relapse, mainly for those with alcohol and cocaine abuse. Alcohol and cocaine use are known to be associated with faster CD4 decline and poorer VL control [29, 38]. Therefore, people with substance use disorders are an important at-risk group among PLWH.

HIV disease progression and immunodeficiency (indicated by CD4 decline) are largely prevented by early initiation of treatment [30] and sustaining an undetectable VL [31]. Our findings suggest that HIV treatment could be a powerful mechanism linking immune preservation and perceived CL self, because the trend towards CD4 preservation was explained by successful HIV treatment. If CL towards self and others predicts a better immunological outcome, this could be indirectly through treatment adherence rather than a direct psychoimmunological process. However, our own research suggests a direct psychoimmunological pathway for spirituality. In the same sample that we used to examine CL, we found that an increase in spirituality after diagnosis [16], a positive view of God [12], and spiritual coping [18, 37] predicted slower CD4 decline and better VL control above and beyond the effect of positive health behaviors. In fact, we found that spirituality was a stronger predictor of health than absence of depression, which was the most powerful psychological predictor [12, 15, 16]. Potentially, *openness and receptivity for spirituality* is an essential criterion for CL to benefit health, although it was one of the least prominent criteria for the perceived benefit of CL in our qualitative analysis. Those results propose that *openness and receptiveness for spirituality* as a criterion for the health benefit of CL warrants further study. This dovetails with concept based on Buddhist thinking of self-compassion of Neff [39], consisting of three main components: self-kindness versus self-judgment, sense common humanity versus isolation versus, and mindfulness versus overidentification. This spirituality based concept of self-compassion predicted physical and psychological well-being in college students [40]. 

Our study suggests that perceived CL towards self predicts better treatment success and lower HIV transmission risk. There is also a trend towards slower HIV disease progression among those rating high on CL self, which is explained by better treatment success. Most importantly, CL towards self no longer predicts undetectable VL over time after controlling for differences in ratings of CL receiving. This may indicate that for those lacking CL self, receiving CL from others potentially protects against cumulative risk of treatment failure and HIV transmission.

### 4.5. Limitations

The main limitation of this study is that the interviews were not initially designed to measure CL, although with very few exceptions, we had enough information for our codings and ratings. In addition, there was a cohort effect where PLWH who died or did not feel well enough to visit our study site may have produced a bias towards positive health behavior and health. A positive self-report bias was also likely for health behaviors. In addition, this study excluded people with active substance use disorder at entry. Furthermore, the results regarding the health behaviors of PLWH in South Florida cannot be generalized to other populations or cultural contexts. Nevertheless, the advantage of studying the relationship between CL and health in PLWH is that there are biological surrogate markers of health behavior and immune function allowing us to generate knowledge on the link between CL and health that may potentially be important for other diseases as well. 

## 5. Conclusions

In summary, *valuing/empowering* and *cognitive understanding* appear to be essential criteria for CL to be perceived as beneficial. Receiving CL is more beneficial for people if it is perceived as given out of free of choice. Perceived CL towards self is highly intercorrelated with giving and receiving other-centered CL. According to our rating, those who do not provide CL to themselves are also least likely to receive CL from others, including health care professionals. Future studies should examine whether health care professionals may prevent the cumulative risk of treatment failure and HIV transmission by walking the extra mile for those patients that lack self-centered CL. According to our ratings, CL towards self forms the basis of giving CL to others and being open to receive CL from others. Most importantly, self-reports of giving CL towards self and others ultimately predict positive biological outcomes over 4 years, such as achieving a cumulative undetectable VL that reduces both HIV disease progression and transmission. The analysis of the association between CL and longevity is forthcoming.

## Figures and Tables

**Table 1:** Five criteria of compassionate love (CL definitions and examples) for each component (giving, receiving, and self).

Criterion	Definition	Example-giving	Example-receiving	Example-self
Free choice	Making a deliberate choice to give oneself for the good of the other/self without having any specific reason or obligation to do so.	“Teaching is what I am supposed to be doing and this is my way of giving back” describes someone's passion for teaching.	“He's there for me always” states someone, who experiences the friend of his diseased father as a “real friend” and truly helpful.	“I decided I have to do something or I'm going to get really sick” describes a participants' reaction to the deaths of many people from AIDS.

Cognitive understanding	The ability to understand the situation, the other, and oneself. This requires the ability to distinguish between the needs, feelings, and wants of the other and oneself.	“I am watching and I am an integral part of people's lives, changing their lives and adjusting them.” describes a teacher. “The kids believe in me because I am very honest”	A HIV-positive partner offers a special insight. “We can talk about anything and everything. He lets me know if I'm doing something right or wrong.”	“I am feeling so good now” rejoices a participant “Little by little it (engagement in psychotherapy) was helping me to understand me, where I'm coming from, and to understand better the others.”

Valuing/empowering	Expressing respect and love for the other/self rather than pity. Central criterion is the enhancement of the recipient's self-efficacy and development.	After being diagnosed with HIV, participant founded a support group aiming “to provide emotional, educational, and social support to the HIV community.”	Participant describes the help of her new boyfriend as follows: “He boosts my self-esteem and is intellectually challenging.”	“It's a challenge, this changes all of your life”, a participant describes his benefit seeking from HIV by using different empowering resources, for example, psychotherapy and education.

Openness and receptivity for spirituality	The spiritual awareness of being part of something important beyond oneself and feeling connected to a higher presence. Being open and receptive for the so-called “inspired” quality of CL.	Someone who believes in reincarnation and karma states “There is a lesson in everyone's lifetime. Now (after HIV diagnosis) I know my lesson. I'm here to help others walk the path, to help them get through this. We all have a purpose of giving back to the universe.”	Participant feels really close to “the Lord” and participant is religious, engaged in different church activities and feels empowered by her church: “A minister in the church put me on his prayer line.”	Despite multimorbidity, someone is full of hope and confidence: “Because I've accepted this and I have hope and faith in God.”

Response of the heart	The affective and emotional dimension of CL. Empathy motivates to help others/self.	A woman remembers the first moments after being diagnosed with HIV-diagnosis: “There was no place for me to go.” To help others with the same destiny, she founded a support group.	Participant's brother was an alcoholic but is sober now and supports his brother: “Matter of fact he came by to sit in on one of my AA meetings.”	“When you see the despair and depths of cruelty people have gone through to survive, then we should be so thankful” a woman describes her gratitude and satisfaction “to have a decent place to live, food, just the basics that most Americans take for granted.”

**Table 2:** Compassionate love components (CL giving, receiving, and self): Rating, definition, and anchor examples.

Rating and definition	Example-giving	Example-receiving*	Example-self
1 CL negative or harmful	A participant, who does not disclose his HIV status and has unprotected sex, describes: “I feel like just having sex with him just to give it (HIV) to him because of the way he used to talk about people with it.”	Someone perceives that nobody is helpful to her. She felt bothered by her family staying at her house: “I already have my own stress, and then I'm taking on their stress”	Participant got HIV as a child from a blood transfusion after an accident. He blames his mother for being HIV-positive because she did not let him die after the accident. To end living with HIV, he tried to commit suicide.

2 No expression of CL	“The loneliness was nearly unbearable” stated someone living in social isolation, spending his days with TV, daydreaming, and drinking, avoiding human interaction	“I have myself and God. I rely on me, my instincts, my intellect,” explains an engaged teacher, who feels that he has “not really” someone who is helpful to him.	A heavy depressed smoker feels overwhelmed by his life, HIV, and taking care of his health: “I've become hopeless, more frustrated, I'm not healthy. I'm not like I used to be. I got fat.”

3 CL attempted but not beneficial	“I want to tell my mom but then I don't want to hurt her heart” explains someone who keeps “important things, like the HIV, locked up.”	A boyfriend's double-edged reaction to her diagnosis showing empathy and fear. First, he promised: “Baby I will be there for you. Don't worry. I love you.” But in daily life she perceived his fear. “He would steam out the shower after me and make me use my own glass and plate. I felt very dirty and very isolated, like a porcelain fragile doll.”	Someone “thinking positive” and “talking to God everyday” believes “He [God] has the power to heal.” On the other hand he struggles with substance use and medication adherence.

4 CL beneficial but not empowering	“I'm keeping myself up and healthy, on account of her”, explains a mother, who sees her main motivation to live in her daughter.	“I couldn't move! I woke up and I was just sick. So he and his sister's took care of me.” described someone who suffered from a severe flu. Another participant receives financially support from her sister: “She just walks up to me, gives me checks,” but there is no empowering relation.	A woman describes her positive self-affirmation: “I had to look in a mirror and say I love myself and other people still love me and they're not afraid of me.”

5 CL beneficial and empowering	Participant who changed his life after being diagnosed. While previously substance abuse was his way to deal with problems, nowadays his most considered coping strategy is to “establishing meaningful friendships and relationships with people and I'm doing service. I'm giving back some of the support and love that was given to me during my time of need” “I'm always talking to people and helping them with connecting with meeting s and that sort of thing.”	A participant feels motivated by his brother: “He's really concerned about me because I'm the youngest. He tries to encourage me to go to meetings, come to church, hang around positive people.” Just after her diagnosis, someone met another HIV-positive woman for founding a support group. “She was like my mentor. She was empowering me.”	“I try to lead the most positive life I can, eating right, not worrying so much, not letting a lot of things stress me out, and always staying happy,” explains a woman's empowering optimism: “I'm trying to get a house built on some land and getting married.”

6 CL is a central element in the person's life*	The founder of a HIV support group describes herself as a “workaholic,” spending all her energy on the project. “This is my life and there are so many people who need me.”		“I think that my house is very therapeutic for me” stated a woman who installed a Jacuzzi in her backyard. She centers her life on her wellbeing, starting work after 1 pm so that she can rest.

*Interviews did not provide information to rate centrality in CL receiving.

**Table 3:** Compassionate love (CL giving, receiving, and self) as predictor of cumulative undetectable viral load (VL) and CD4 cell preservation over 4 years.

Predictor	Prediction to^a^	Controlling for	Coefficient	*T *ratio	*n* ^b^	*P* ^c^
CL giving	Cumulative undetectable VL		0.011	1.055	156	.147
	CD4 cell preservation		−0.191	−0.552	156	.291

CL receiving	Cumulative undetectable VL		<0.001	0.023	168	.491
	CD4 cell preservation		−0.777	−0.824	168	.206

CL self	Cumulative undetectable VL		0.010	2.841	166	.003
		Substance use disorder	0.008	2.390	159	.009
		CL giving	0.012	3.068	152	.002
		CL receiving	0.002	0.470	164	.319
	CD4 cell preservation		0.495	1.220	166	.112
		Substance use disorder	0.424	1.049	159	.148
		Cumulative undetectable VL	0.017	0.080	166	.468
		CL giving	0.766	1.671	152	.048
		CL receiving	0.754	1.586	164	.057

^a^All models are controlled for differences in age, gender, ethnicity, education, CD4 cells, and undetectable VL at entry.

^b^Participants with missing data on CL, substance use disorder, VL and CD4 cells were excluded, DF = *n* − 9.

^c^One-tailed test.

## Abbreviations

CD4: Cell surface receptor that HIV uses for cell entry; CD4 cell decline is a surrogate marker HIV disease progression and immunodeficiency
CL: Compassionate love
HIV: Human immunodeficiency virus
PLWH: People living with HIV
LR: Likelihood ratio
VL: Viral load of HIV, undetectable under taking and adhering to effective HIV treatment
WHO: World Health Organization.
